# Effects of methionyl-methionine dipeptide on fecal microbiota in transition dairy cows

**DOI:** 10.3168/jdsc.2026-1084

**Published:** 2026-09-07

**Authors:** Yue Liu, Ye Xue, Chen Zhang, Min Li, Hangshu Xin, Hongyun Liu

**Affiliations:** 1 Institute of Dairy Science, College of Animal Sciences, Zhejiang University, Hangzhou 310058, China; 2 College of Animal Science and Technology, Northeast Agricultural University, Harbin 150030, China

## Abstract

Transition dairy cows undergo substantial metabolic stress, which increases the demand for methionine, a key amino acid involved in milk synthesis and metabolic regulation. Methionyl-methionine dipeptide is a methionine-containing dipeptide that has been shown to promote milk protein synthesis in vitro and improve milk protein production in vivo. Although the effects of methionine supplementation on ruminal metabolism in dairy cows have been widely studied, the effects of methionyl-methionine dipeptide on the fecal microbiota of transition dairy cows remain poorly characterized. At 28 d prepartum, 45 Holstein cows were blocked by parity and randomly assigned to control (CON; basal diet), unprotected methionyl-methionine dipeptide (MM; basal diet + 8 g/d MM), or rumen-protected methionyl-methionine dipeptide (RPMM; basal diet + 22 g/d RPMM, providing 8 g/d intestinally available MM). At 21 ± 1 d postpartum, 6 representative cows per treatment were selected for fecal sampling and 16S rRNA gene sequencing. Fecal microbial diversity and community structure differed among treatments, whereas the dominant taxa at the phylum and genus levels remained largely consistent. Among the differentially abundant genera, *Turicibacter* was more abundant in the RPMM group than in the CON and MM groups and was positively associated with milk yield, plasma total protein, and plasma total antioxidant status. *Howardella* was less abundant in the RPMM group than in the CON group and was positively associated with plasma BHB and nonesterified fatty acids, but negatively associated with milk protein concentration. Compared with the RPMM group, the MM group had a higher relative abundance of *[Eubacterium]_siraeum_grou*p and *Colidextribacter* and a lower relative abundance of *Frisingicoccus*, with *Colidextribacter* positively associated with milk protein concentration and plasma glucose concentration. Overall, supplementation with MM or RPMM was associated with distinct shifts in the fecal microbiota, with RPMM-related taxa showing closer associations with lactation performance and plasma metabolic parameters. These findings advance our understanding of the links between methionyl-methionine supplementation, fecal microbiota, and metabolic adaptation in transition dairy cows.

## Graphical Abstract

**Figure:**
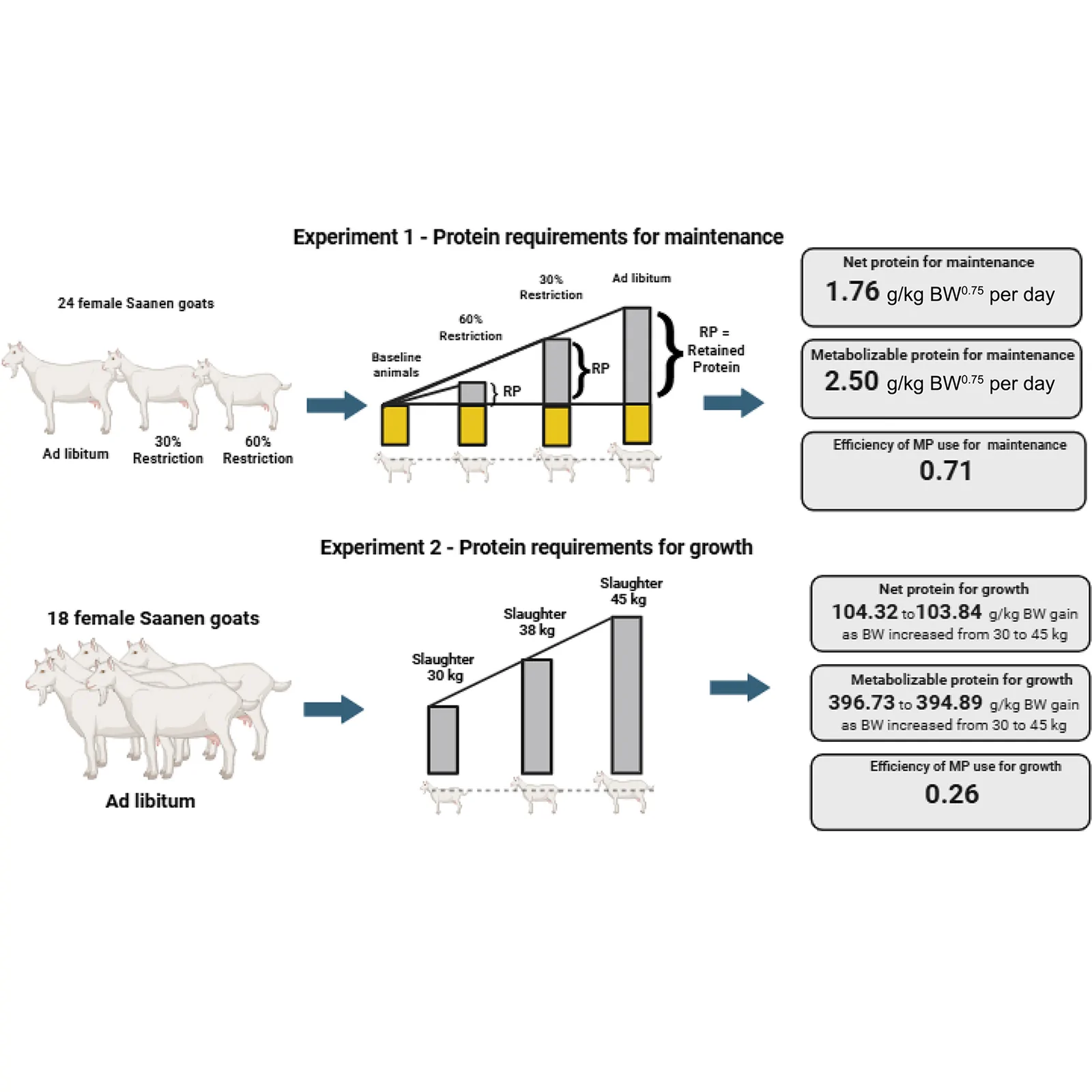

The transition period, typically defined as the period from 21 d before to 21 d after calving, is a critical stage for dairy cows (Drackley, 1999; Caixeta and Omontese, 2021). During this phase, cows commonly experience negative energy balance, oxidative stress, and inflammatory responses, which are associated with impaired health and lactation performance (Mezzetti et al., 2021; Toledo et al., 2021). In dairy cows, feed intake can shape digestive microbial ecosystems, including both the rumen and hindgut (Bach et al., 2019; Lin et al., 2024). Additionally, growing evidence suggests that alterations in the hindgut microbiota are closely associated with the metabolic and immune homeostasis of transition dairy cows and may contribute to their physiological adaptation to early lactation (Tröscher-Mußotter et al., 2021; Luo et al., 2024; Wang et al., 2025).

Methionine is a limiting amino acid in dairy cows and plays essential roles in protein synthesis, methyl-group metabolism, and antioxidant capacity (Sun et al., 2016; Batistel et al., 2018). Methionyl-methionine, a dipeptide composed of 2 Met residues, can be taken up intact from the intestinal lumen and absorbed more rapidly than an equivalent amount of free Met in rats (Lis et al., 1972). Moreover, methionyl-methionine has been shown to increase α_S1_-casein expression in bovine mammary epithelial cells and to promote α_S1_-casein synthesis in bovine mammary gland explants, with *JAK2-STAT5* and *mTOR* signaling pathways implicated in the latter response (Wu et al., 2007; Yang et al., 2015).

Compared with unprotected methionyl-methionine dipeptide (**MM**), rumen-protected methionyl-methionine dipeptide (**RPMM**) is designed to reduce ruminal degradation and increase the postruminal delivery of MM. Our previous study in mid-lactation cows showed that RPMM increased milk yield, milk protein concentration, and milk protein yield (Wei et al., 2024). Consistent with these findings, our subsequent study during the transition period showed that cows supplemented with RPMM had significantly higher ECM yield and milk protein yield than those supplemented with MM (data not shown). These findings raise the question of whether the favorable lactation performance observed with RPMM supplementation is accompanied by distinct changes in the fecal microbiota. Accordingly, we hypothesized that MM and RPMM supplementation would alter the fecal microbiota of transition dairy cows and that the lactation response to RPMM would be linked to changes in specific fecal bacterial taxa.

The experiment was conducted on a commercial dairy farm (Hangzhou, China), and all procedures were approved by the Animal Welfare Committee of Zhejiang University (Hangzhou, China; protocol no. 22955). The experiment began 28 d before calving, included a 7-d acclimation period, and lasted until 21 d after calving. Forty-five Holstein cows were blocked by parity and randomly assigned to 1 of 3 treatments: control (**CON**; basal diet), MM (basal diet + 8 g/d MM), and RPMM (basal diet + 22 g/d RPMM). The MM product was provided by Evonik Ltd. (Essen, Germany), and the rumen-protective coating was performed by King Techina Co. Ltd. (Hangzhou, China). The RPMM product contained 50% MM and had estimated ruminal bypass and intestinal release rates of 90% and 80%, respectively. Therefore, the RPMM dose was set at 22 g/d to provide ∼8 g/d of intestinally available MM.

Cows were housed in group pens according to treatment, and the basal TMR was offered 3 times per day (0700, 1430, and 2100 h). After morning milking, the complete daily dose of MM or RPMM was mixed with a small amount of fresh TMR and offered individually while cows were restrained in individual headlocks; trained personnel confirmed that the supplement-containing portion was completely consumed and that no supplement-containing TMR remained before release, thereby preventing cross-consumption. At 21 ± 1 d postpartum, 6 cows per treatment were selected for microbiota sequencing based on parity, with milk yield and blood parameters close to the corresponding treatment means, thereby reducing the potential influence of extreme individuals. The V4 region of the bacterial 16S rRNA gene was amplified using primers 515F (5′-GTGCCAGCMGCCGCGG-3′) and 806R (5′-GGACTACHVGGGTWTCTAAT-3′). Amplicons were sequenced on the Illumina platform (Illumina Inc.). High-quality sequences were then denoised using DADA2 (https://benjjneb.github.io/dada2) to generate amplicon sequence variants (**ASV**).

Alpha diversity indices were calculated using Mothur (v. 1.30.2, https://mothur.org/), and differences among treatments were tested using the Kruskal–Wallis test. Beta diversity was visualized using principal component analysis (**PCA**), and treatment differences were evaluated using analysis of similarities (**ANOSIM**). Differentially abundant genera were identified using the Kruskal–Wallis test with false discovery rate correction. Spearman correlation analysis was performed to assess associations of differentially abundant genera with lactation performance and plasma metabolic parameters. Except for the α-diversity analyses, all other statistical analyses were conducted in R (v. 3.3.1, https://www.r-project.org/).

In this study, the Simpson index differed among treatments, with the highest value observed in the RPMM group and the lowest in the MM group (Figure 1A). The PCA plot showed separation of fecal microbial communities among treatments, and ANOSIM indicated significant differences in fecal microbial community structure (Figure 1B). At the phylum level, *Bacillota* and *Bacteroidota* were the dominant taxa across all groups, whereas the dominant genera were largely consistent across treatments (Figure 1C–D). In transition dairy cows, Arnalot et al. (2025) also observed shifts in fecal bacterial diversity and taxonomic composition around calving, with *Bacillota* and *Bacteroidota* remaining the predominant phyla. These results suggest that fecal microbial diversity and community structure differed among treatments, whereas the dominant taxonomic composition remained relatively stable. Therefore, the observed differences may primarily reflect shifts in the relative abundance of specific bacterial taxa.

**Figure 1:**
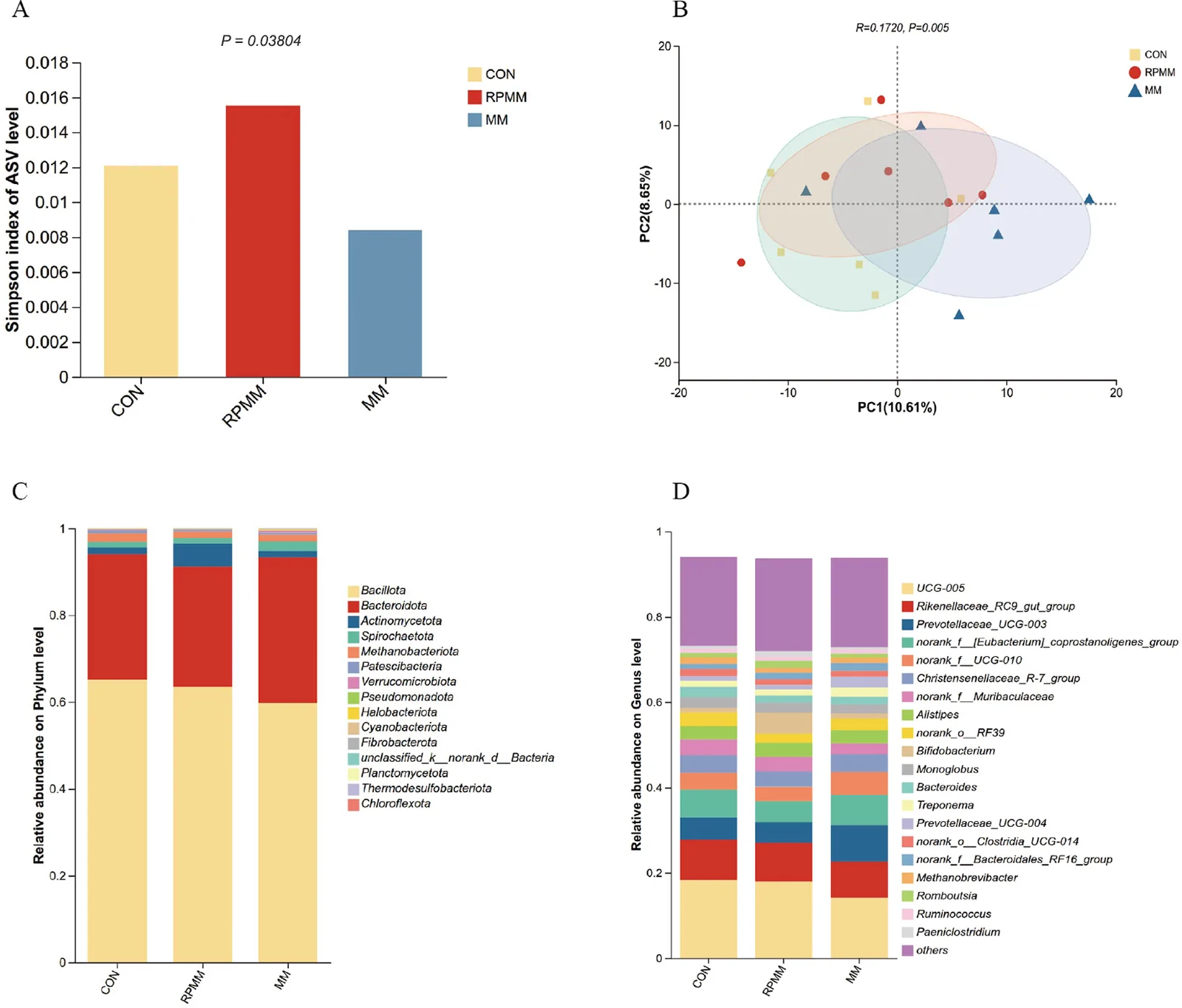
Diversity and taxonomic composition of fecal microbiota in transition dairy cows receiving different treatments. (A) Simpson index of α-diversity; (B) principal component analysis (PCA); (C) relative abundance of microbiota at the phylum level; (D) relative abundance of microbiota at the genus level. PC = principal component.

Compared with the RPMM group, the MM group had a higher relative abundance of *[Eubacterium]_siraeum_group* and *Colidextribacter*, and a lower relative abundance of *Frisingicoccus* (Figure 2A). Supplementation with MM may influence the fecal microbiota by altering ruminal microbial metabolism and the substrates reaching the hindgut, rather than through direct exposure of hindgut bacteria to intact MM. A study showed that supplementation with a Met analog increased ruminal microbial nitrogen flow and altered the relative abundance of *Faecalibacterium*, *Eubacterium*, and several *Prevotella* taxa in lactating dairy cows (Lee et al., 2015). *[Eubacterium]_siraeum_group* was enriched in the intestinal microbiota of wild zokors whose microbial communities showed greater predicted capacity for carbohydrate metabolism and cellulose degradation (Zou et al., 2021). Therefore, its higher abundance in the MM group may indicate altered availability of plant-derived fermentable substrates in the hindgut following ruminal utilization of unprotected MM, rather than a direct stimulatory effect of MM on this taxon. In contrast, *Frisingicoccus* was less abundant in the MM group than in the RPMM group. In transition dairy cows, its abundance increased during the development of hyperketonemia and was positively associated with circulating branched-chain amino acids, blood BHB, and microbial genes related to ketogenesis (Luo et al., 2024). These findings suggest that *Frisingicoccus* may be sensitive to changes in host amino acid and energy metabolism. Therefore, its higher abundance in the RPMM group may reflect the greater expected postruminal MM supply and the resulting differences in host hindgut amino acid metabolism compared with MM. However, this pattern should not be interpreted as necessarily beneficial, and the direct role of *Frisingicoccus* remains unclear.

**Figure 2:**
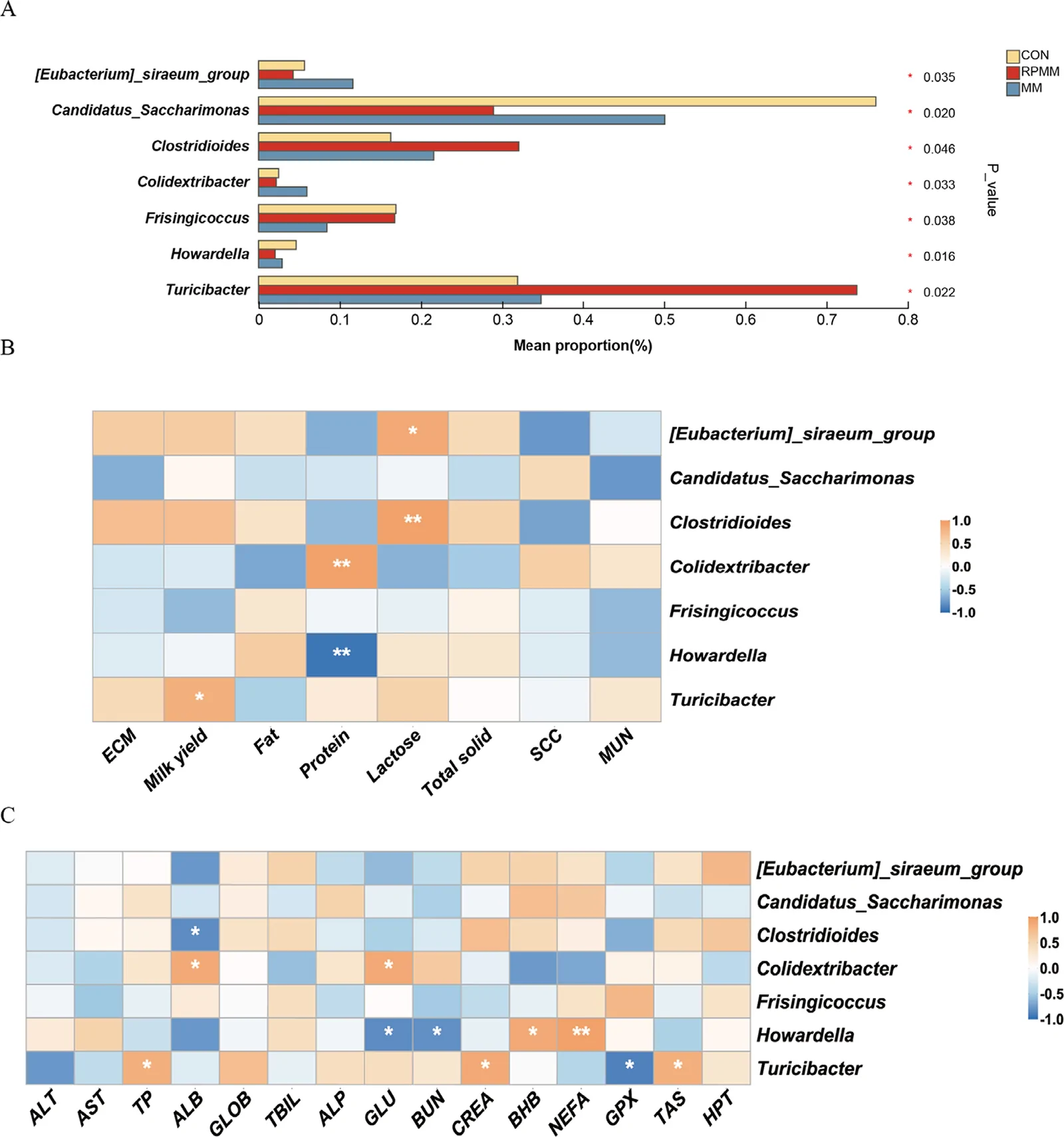
Differentially abundant genera and their associations with lactation performance and plasma metabolic parameters in transition dairy cows. (A) Differentially abundant genera; (B) associations between differentially abundant genera and lactation performance and milk composition parameters; (C) associations between differentially abundant genera and plasma metabolic parameters. In panel B, units are kg/d for ECM and milk yield, % for milk fat, protein, lactose, and total solids concentrations, and × 10^3^ cells/mL for SCC. ALB = albumin, g/L; ALT = alanine aminotransferase, U/L; ALP = alkaline phosphatase, U/L; AST = aspartate aminotransferase, U/L; CREA = creatinine, μmol/L; GLU = glucose, mmol/L; GLOB = globulin, g/L; GPX = glutathione peroxidase, U/L; HPT = haptoglobin, mg/mL; NEFA = nonesterified fatty acids, μmol/L; TBIL = total bilirubin, μmol/L; TAS = total antioxidant status, mmol/L; TP = total protein, g/L. *P < 0.05; **P < 0.01.

*Turicibacter* had a higher relative abundance in the RPMM group than in the CON and MM groups and was positively associated with milk yield, plasma total protein, and plasma total antioxidant status (Figure 2A–C). Previous studies have linked *Turicibacter* to host lipid and bile acid metabolism and immune-related parameters (Hamada et al., 2023; Lynch et al., 2023), suggesting that its higher relative abundance in the RPMM group may be related to differences in host metabolic and antioxidant status. Rumen-protected Met supplementation has also been reported to alleviate oxidative stress and inflammation in transition dairy cows (Batistel et al., 2018). In the small intestine, MM may be hydrolyzed to free Met or partly absorbed as an intact dipeptide (Xu et al., 2018). Absorbed Met can support protein synthesis and enter transmethylation and transsulfuration pathways, thereby affecting host nitrogen utilization and redox status (Zhou et al., 2018; Potts et al., 2023). Given the positive association between *Turicibacter* and plasma total antioxidant status observed in the present study, these host responses may partly contribute to the higher abundance of *Turicibacter*, although a direct link remains to be confirmed.

Additionally, *Howardella* had a lower relative abundance in the RPMM group than in the CON group and was negatively associated with milk protein concentration but positively associated with plasma BHB and nonesterified fatty acids (Figure 2A–C). Higher ruminal abundance of *Howardella* has been associated with lower DMI in lactating Holstein cows, possibly because reduced feed intake increases ruminal urea recycling and favors ureolytic taxa such as *Howardella*, although urea recycling was not directly measured (Monteiro et al., 2022). In the present study, the associations with BHB, nonesterified fatty acids, and milk protein suggest that *Howardella* may be linked to metabolic adaptation during the transition period. Its lower abundance in the RPMM group may also reflect altered availability of urea or other nitrogenous substrates following improved postruminal Met utilization.

*Colidextribacter* was more abundant in the MM group and was positively associated with milk protein concentration and plasma glucose (**GLU**) concentration. Because unprotected MM is more readily digested in the rumen, MM supplementation may alter ruminal fermentation and the substrates subsequently reaching the hindgut, thereby creating a microbial environment that favors *Colidextribacter*. Previous research reported a negative association between *Colidextribacter* and the ruminal acetate-to-propionate ratio, suggesting a possible link with glucogenic substrate availability (Hao et al., 2024), which may partly explain its associations with GLU and milk protein concentration. However, the higher abundance of *Colidextribacter* in the MM group does not necessarily explain the lactation response to MM or RPMM. Its enrichment may mainly reflect changes in gastrointestinal fermentation and substrate flow induced by unprotected MM, whereas the greater milk protein yield with RPMM may be more directly related to increased postruminal MM availability. Therefore, the treatment difference in *Colidextribacter* abundance and its positive association with milk protein concentration may represent different responses to MM supplementation.

Overall, MM and RPMM supplementation were associated with distinct shifts in the fecal microbiota in transition dairy cows. Compared with MM, RPMM-related microbial changes showed closer associations with lactation performance and plasma metabolic parameters, suggesting that RPMM-associated hindgut bacterial taxa may be linked to more favorable metabolic adaptation during the transition period. However, gastrointestinal MM and Met availability, hindgut metabolites, and microbial functions were not directly measured. Therefore, the mechanisms underlying these associations remain to be confirmed. These findings may help guide future studies on how selected fecal bacterial taxa are associated with metabolic adaptation in transition dairy cows.
